# Combating lead and cadmium exposure with an orally administered chitosan-based chelating polymer

**DOI:** 10.1038/s41598-023-28968-4

**Published:** 2023-02-07

**Authors:** Jordyn Ann Howard, Halyna Kuznietsova, Natalia Dziubenko, Axel Aigle, Marco Natuzzi, Eloise Thomas, Vladimir Lysenko, Laurent David, Thomas Brichart, François Lux, Olivier Tillement

**Affiliations:** 1 MexBrain, 13 Avenue Albert Einstein, 69100 Villeurbanne, France; 2 Institute of Light and Matter, UMR 5306, University of Lyon 1-CNRS, University of Lyon 1, Villeurbanne Cedex, France; 3 Corporation Science Park, Taras Shevchenko National University of Kyiv, 60 Volodymyrska Str., Kyiv, 01033 Ukraine; 4 LAGEPP, Université Claude Bernard Lyon 1, CNRS, UMR5007, Villeurbanne, France; 5 Institut Universitaire de France (IUF), Paris, France; 6 Univ Lyon, Université Claude Bernard Lyon 1, INSA de Lyon, Université Jean Monet, CNRS, UMR 5223 Ingénierie des Matériaux Polymères (IMP), 15 Bd A. Latarjet, 69622 Villeurbanne Cedex, France

**Keywords:** Drug discovery, Chemistry

## Abstract

Heavy metals present a threat to human health, even at minimal concentrations within the body. One source of exposure is due to the consumption of low-level contaminated foodstuff and water. Lead and cadmium have been shown to be absorbed by and accumulate within organs like the kidneys and liver, and they have also been associated to many diseases including cardiovascular disease and kidney dysfunction as well as developmental disorders and neurodegenerative diseases. Since this contamination of lead and cadmium is found worldwide, limiting the exposure is complicated and novel strategies are required to prevent the absorption and accumulation of these metals by forcing their elimination. In this study, a DOTAGA-functionalized chitosan polymer is evaluated for this preventative strategy. It shows promising results when orally administered in mice to force the elimination and negate the toxic effects of lead and cadmium found within foodstuff.

## Introduction

Metals have a complex and ubiquitous role in living organisms. Oligo-metals, like iron, manganese, copper, and zinc are considered essential to life, and their concentrations are finely regulated by the body to attain an optimal biological function^1^. On the other hand, some metals, like lead and cadmium, have no known biological role. Classified among the 10 most dangerous chemicals by WHO and as Group 2b and 1b carcinogens by the International Agency for Research on Cancer, for lead and cadmium respectively, the dangers of these metals are well known^2,3^. There exists no acceptable concentration within humans; even trace concentrations have been linked to severe health risks, including, but not limited to, neurodegeneration and neurodegenerative diseases like Parkinson’s disease, cardiovascular disease, kidney failure and chronic kidney disease (CKD), stunted fertility in men, miscarriages, and abnormal development in fetus and children^4–7^. Although the mechanisms are not yet completely understood, the strong affinities of lead and cadmium for sulfur-containing biomolecules in particular, as well as those containing nitrogen and oxygen to a lesser amount^8^, their ability to displace oligo-metals^9,10^, and their role in the generation of reactive oxygen species (ROS) have been proposed as the underlying causes of such pathologies^6,9–14^.

These ubiquitous heavy metals are found within our environment as consequences of the human industrialization (waste disposal, pollution from factories, etc.) which humans are exposed to on a daily basis^15–17^. While tobacco products are the largest source of exposure for the smoking population, foodstuff (animals, crops, etc.) and water remain the largest source of contamination for the majority of the non-smoking population^5,17^. There are many legislative groups (regionally, nationally, and internationally) that attempt to protect humans by regulating our exposure to heavy metals as much as possible^18^. It is particularly the case for high income countries for lead since 1970 with limits on paint, gasoline, food cans or water utilities^19^. Unfortunately due to the inheritance from the past, lead and cadmium contamination is still an important issue even in high income countries^20,21^. Unfortunately, the average population still consumes a few micrograms of lead and cadmium per day from contamination foodstuff and water^22,23^. Studies have directly linked the intake from foodstuff to up to a 3-fold increase in cancer mortality and up to a 4-fold increase in CKD risk^24^.

Heavy metals from contaminated foodstuff and water enter the body through the digestive tract where they are absorbed into the bloodstream through the intestinal membrane. Once these heavy metals are integrated within the body, the majority are eliminated by the renal system within the urine, but a smaller portion remains within the bloodstream and/or is re-absorbed by the kidneys and eventually accumulates within the liver, kidneys, and bones. Certain pathologies, like CKD, as well as aging can lead to a reduced efficiency of the body’s natural elimination pathways, therefore increasing the accumulation of these heavy metals resulting in an increase in toxicity. Lead and cadmium have been identified as nephrotoxic, leading to a decrease in the glomerulus filtration rate (GFR) and overall elimination efficiency^17,25,26^. Therefore a vicious cycle is provoked: decreased natural elimination due to the pre-existing pathology or age leads to a more localized accumulation of heavy metals within the kidneys, which further decreases the filtration/elimination rate, etc.

In the case of heavy metal poisoning (greater than 200 µg L^−1^ of lead or cadmium within the blood), chelation therapy can be proposed to help decrease these high blood concentrations by forcing the elimination of the metals, which is most commonly performed with intravenous treatments of edetate calcium disodium (CaNa_2_EDTA) as well as 2,3-dimercaptosuccinic acid (DMSA) and sodium 2,3-dimercaptopropane 1-sulfonate (DMPS)^20,27^.

The majority of lead and cadmium contaminations is relatively low-level, within the parts per billion range (0.1–100 µg L^−1^), and is no longer considered an “acute” heavy metal poisoning. For this population, the adverse effects of chelation therapy (hepatotoxicity, hypotension, coagulation disorders, etc.) outweigh the minimal therapeutic chelation efficiency observed with these “chronic” concentrations and is not recommended^27–29^. Some clinical studies within specific, highly sensitive populations, such as cardiovascular disease (CVD) and diabetes, have shown positive results with the use of long-term chelation therapy^30^, however, the only real recommended therapy for those with low, non-acute blood levels is to reduce one’s exposure to the source of contamination. This is difficult to achieve when the primary source of exposure is foodstuff and water. Even with a top-tier selection of products, it is nearly impossible to avoid the contamination of heavy metals completely, which can have severe implications for patients already suffering from CKD and/or End Stage Renal Disease (ESRD), CVD, neurodegenerative diseases, etc.

This study aims to evaluate the effectiveness of a DOTAGA-functionalized chitosan polymer, which can be easily orally administered, to neutralize traces of heavy metals originating from consumed foodstuff and/or water. More specifically, this polymer will target the heavy metal cations that can easily penetrate the intestinal membrane. The hypothesis of this paper is to show that through the specific targeting and chelation of these heavy metal cations within the digestive tract with a polymer of sufficient size, one can provide a prevention, or at least a minimization, of the absorption of these heavy metals and therefore their associated toxic effects on the biological system.

Based on the well-known and efficient chelation of lead and cadmium by DOTA in trace concentrations and even complex media (complex formation constants, log(β)_DOTA_ = 22.69 and 21.3, respectively, See Table S0)^31,32^, its closely related derivative, DOTAGA, was chosen for functionalization of the polymer. Its additional grafting arm provides an intact DOTA moiety for complexation after the functionalization addition to the polymer. Chitosan is a biocompatible polymer that is primarily extracted from crustacean shells and fungi. It is known for its current use within the diabetic population for weight control, where it can be consumed on the gram scale^33^. This polymer motivates strong research interest for its use in drug delivery, tumor targeting, and vaccine delivery^34^. Using a sufficiently high molar mass, the polymer described in this study will not only remain within the digestive tract, but will retain any chelated heavy metals with it before its elimination via the fecal route. With this strategy, we hope to provide a novel strategy to counter low-level heavy metal exposure that can be implemented within a wide range of patients.

In this work, the characterization of lead and cadmium chelation by the polymer and in vivo mouse studies are presented.

## Results

### Synthesis and characterization of Chitosan@DOTAGA

**Figure 1:**
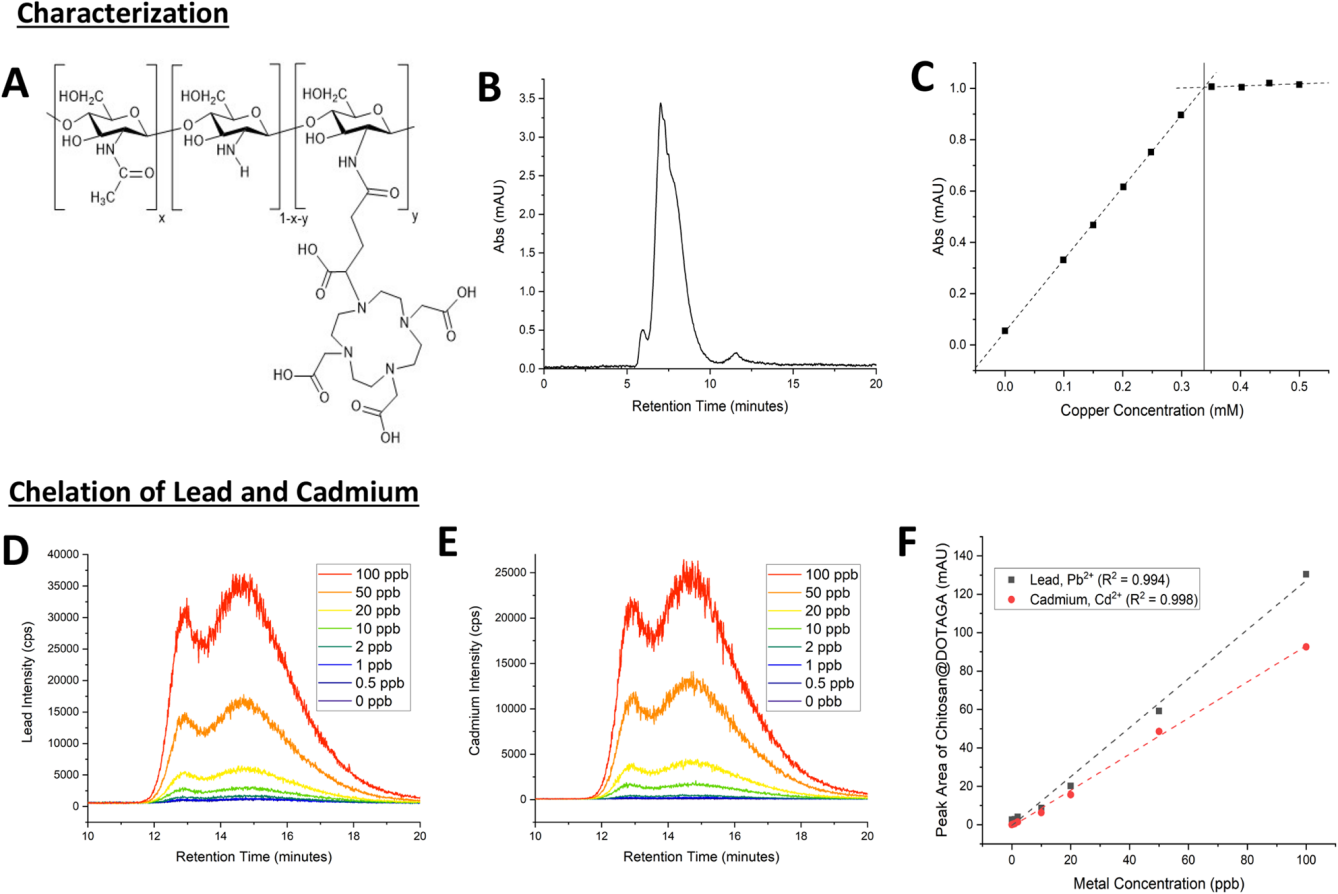
(**A**) Representation of re-acetylated chitosan functionalized by DOTAGA. Chitosan@DOTAGA where x corresponds to the acetylation ratio and y corresponds to the DOTAGA grafted ratio. (**B**) SEC HPLC-UC elution diagram of 10 g L^−1^ Chitosan@DOTAGA in aqueous medium in absence of Cu(II) using detection at 295 nm. The peak at 7 min corresponds to the Chitosan@DOTAGA polymer, while the smaller peak at 11 min corresponds to residual, un-grafted DOTAGA. (**C**) Dosage of a solution of 1 g L^−1^ of Chitosan@DOTAGA by Cu(II) using SEC HPLC–UV. The integration of the polymer peak at 7 min was performed for each added concentration of copper providing a linear relationship until saturation of all available DOTAGA. (D) SEC HPLC–MS of 0.1 g L^−1^ of Chitosan@DOTAGA in the presence of varying Pb(II) concentrations. (**E**) SEC HPLC–MS of 0.1 g L^−1^ of Chitosan@DOTAGA in the presence of varying Cd(II) concentrations. This method of analysis employs a slower flow rate (0.4 mL min^−1^ compared to 0.8 mL min^−1^) than the previous, providing a longer Chitosan@DOTAGA retention time of 14.6 min, which is in agreement with the previous retention time of 7 min. Both peaks visualized in this method are representative of the same species, Chitosan@DOTAGA. Lead and cadmium chelation by Chitosan@DOTAGA follows a similar trend. (**F**) Dosage of a solution of 0.1 g L^−1^ of Chitosan@DOTAGA by Pb(II) and Cd(II) using detection of isotopic masses. The integration of the polymer peaks in (**D**) and (**E**) were performed for each metal concentration providing a linear relationship. A slight preference of chelation can be seen for lead, which is in agreement with the complexation coefficients.

Functionalized chitosan (Chitosan@DOTAGA), represented in Fig. 1A, is synthesized in two-steps, following the same scheme presented by Natuzzi et al., 2021. Initially, the chitosan is re-acetylated with acetic anhydride in a water/1,2-propanediol mixture to improve the polymer’s solubility. ^1^H NMR is employed to determine the degree of acetylation: 30 ± 2% (x = 0.3). After re-acetylation, the functionalization step is performed by the introduction of DOTAGA-anhydride (1,4,7,10-tetra-azacyclododecane-1-glutaric anhydride-4,7,10-triacetic acid) to the solution. The polymer is purified by tangential filtration (cut-off: 100 kDa) and freeze-dried for long-term storage. The purity of the polymer is then qualitatively and quantitatively assessed by size exclusion chromatography (SEC) coupled with UltraViolet–Visible detection (SEC HPLC–UV).

The chromatogram is presented in Fig. 1B. The polymer has a retention time of 7 min due to its large size, and the smaller, not grafted DOTAGA has a retention time of 11 min. The purity of the product was determined to be 97.7% by peak integration.

The amount of DOTAGA successfully grafted on the polymer was determined using copper chelation as described previously by Natuzzi et al.^35^. Briefly, various solutions of the polymer are prepared with a constant polymer concentration and increasing copper concentrations. Copper is chosen due to DOTAGA’s ability to reliably chelate it (log(β)_DOTA_ = 22.3)^32^ and its absorbance at 295 nm. As the copper is chelated by the polymer, the peak obtained by SEC at 295 nm, which corresponds to the functionalized polymer, continuously increases with the copper concentration until a saturation of all DOTAGA chelating species is achieved, as shown in Fig. 1C. This saturation concentration of copper therefore represents the amount of the chelating agent successfully grafted onto the polymer. The amount of DOTAGA available for chelation on the polymer was determined to be 0.338 mmol g^−1^.

### Characterization of lead and cadmium chelation by Chitosan@DOTAGA

The polymer’s chelation abilities were assessed by size exclusion chromatography coupled with mass spectrometry (SEC HPLC–MS) for lead and cadmium isotopes. Increasing concentrations of lead and cadmium were added to a fixed amount of Chitosan@DOTAGA to assess the linearity of heavy metal chelation, like that seen with copper. It can be noted that these samples contained both metals to provide a proof of the dual chelation theory in the presence of both lead and cadmium. Samples exhibiting a polymer concentration of 100 mg L^−1^ and metal concentrations ranging from 0.5 to 100 µg L^−1^ were prepared in MilliQ water. These concentrations provide an excess of DOTAGA in comparison to the metal concentrations.

Indeed, with a grafted DOTAGA concentration of 0.338 mmol.g^−1^ (Fig. 1C), it can be calculated that 1 mg exhibits 3.38 × 10^–7^ mol of available DOTAGA for the chelation of lead and cadmium. DOTAGA is understood to chelate metal ions in a 1:1 ratio (Metal:Ligand, ML) like DOTA^32^. 1 mg of Chitosan@DOTAGA can therefore chelate up to 70 µg of lead (M_w_ = 207.2 g mol^−1^) and 38 µg of cadmium (M_w_ = 112.4 g mol^−1^).

**Figure 2:**
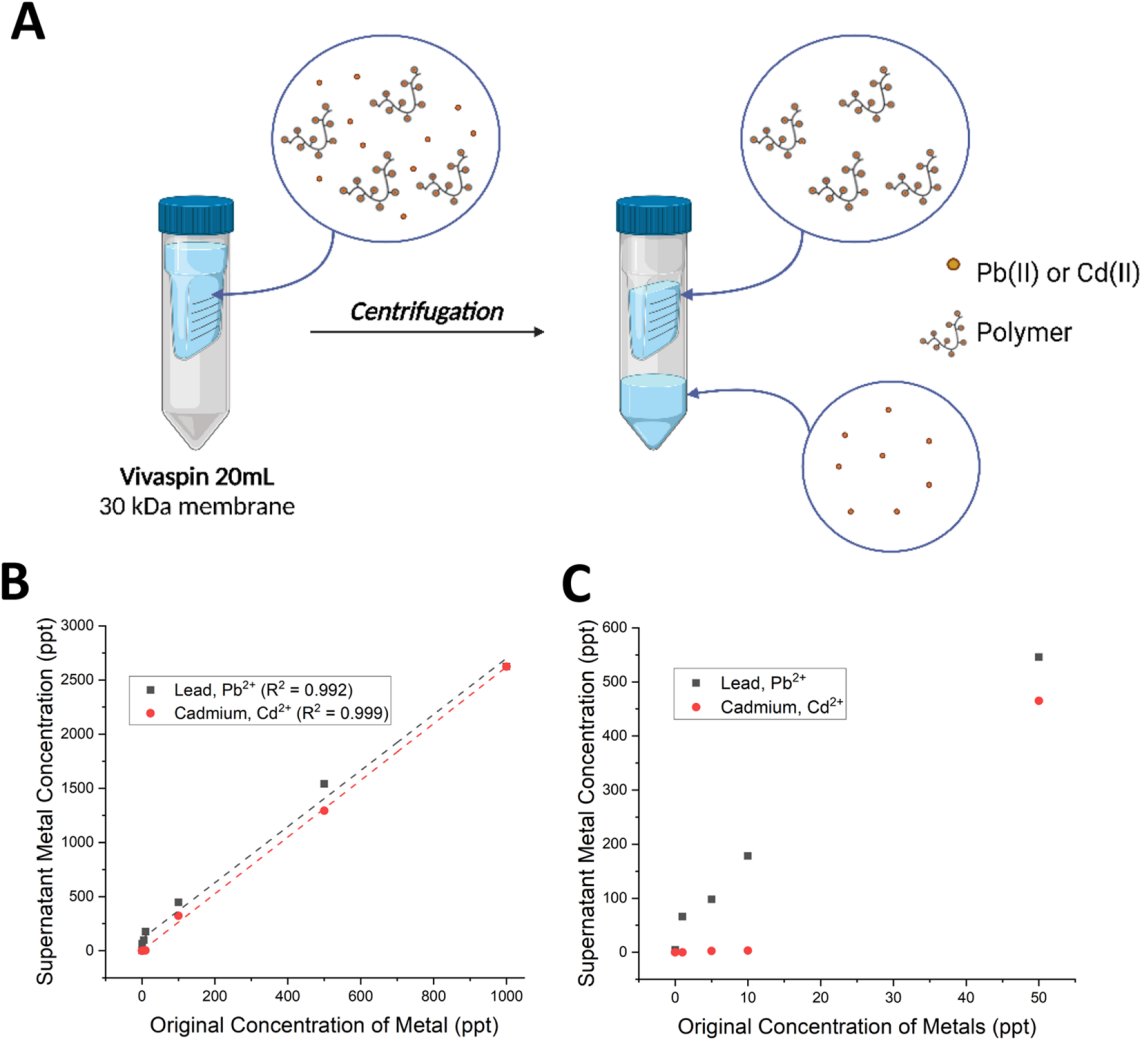
(**A**) Scheme of Vivaspin experiment. The metals that are not chelated pass through the membrane, while chelated metals are bound to the polymer that cannot pass through the membrane. *Image created with BioRender.* (**B**) General linear trend of lead and cadmium chelation by 1 mg L^−1^ Chitosan@DOTAGA. A small polymer concentration is able to target and chelate lead and cadmium within the parts per trillion domain (ppt) in a linear fashion. (**C**) Trace chelation of lead and cadmium by 1 mg L^−1^ Chitosan@DOTAGA. Upon further exploration of the linear trend visualized in (**B**), chelation of lead and cadmium seems to begin within the domain of 10 ng L^−1^ for lead and 50 ng L^−1^ cadmium.

As shown in Fig. 1D–E, Chitosan@DOTAGA does not exhibit any relevant peaks in the absence of metals, but as the concentration of lead or cadmium increases, an equally increasing Chitosan@DOTAGA polymer peak at 14.6 min can be observed. This retention time is correlated and in agreement with the retention time of 7.0 min in Fig. 2B due to the difference in flow rate within the employed methods (0.4 mL min^−1^ for SEC HPLC–MS compared to 0.8 mL min^−1^ for SEC HPLC–UV). The polymer peak in SEC HPLC–MS is seen from 12 and 18 min with two peaks. This is due to its large size, the separation, and the dead volume of the chosen SEC column used, but both of these peaks belong to the same species and are representative of the polymer Chitosan@DOTAGA. The integration therefore considers all of the polymer peak from 12 to 18 min and was performed to assess the relationship between the chelation and increasing metal concentration, which was found to be linear in the case of both lead and cadmium as shown in Fig. 1F. The slightly stronger chelation affinity for lead can also be observed within this experiment which is in agreement with the complexation coefficients (log(β)_DOTA_ = 22.69 for lead and 21.3 for cadmium)^32^.

In separate experimentation, lead and cadmium chelation was assessed by Inductively Coupled Plasma Mass Spectrometry. Samples with varying lead and cadmium concentrations (0–500 ng L^−1^) were employed to understand the chelating abilities of 1 mg L^−1^ polymer prepared in MilliQ water. These samples were centrifuged using Vivaspin 20 Centrifuge Tubes equipped with a 30 kDa membrane (Fig. 2A). Because of the polymer’s large size, it cannot pass through the Vivaspin membrane and will be retained with any chelated metals. If the metals are not chelated, they can freely pass to through the membranes. The original solution as well as the two portions (above (supernatant) and below (undernatant) the membrane) were assessed by ICP-MS and the results are presented in Fig. 2B and C. It was confirmed that in the absence of a chelating polymer the metals pass through the Vivaspin membrane and are only found below.

Chitosan@DOTAGA chelation of lead and cadmium follows a linear trend, even within the domain of trace metal concentrations (part per trillion, ppt or ng L^−1^). Lead chelation appears to be more efficient in this experiment as well providing another experimental agreement with the complexation coefficients for these metals^32^. Based on the observed trends in Fig. 2C, the lower limit of chelation for lead appears to be of the order of 10 ng L^−1^ while cadmium chelation by the polymer appears to be in the domain of 50 ng L^−1^. This is especially interesting considering the dangers of lead and cadmium even at low concentrations.

### In vivo oral administration study of Chitosan@DOTAGA in mice

In vivo proof of concept studies were performed on a mice model to determine the feasibility of the polymer Chitosan@DOTAGA.

**Figure 3:**
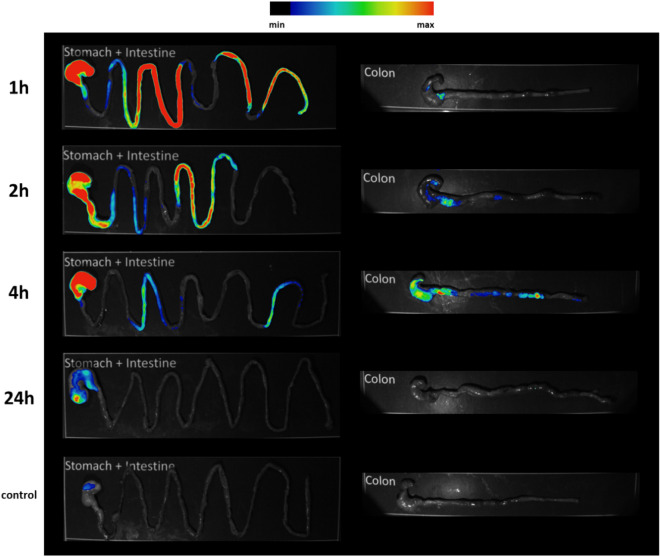
Visualization of Cyanine5.5-labelled Chitosan@DOTAGA over 24 h after oral administration in mice. The Cyanine5.5-labelled Chitosan@DOTAGA can be easily identified within the digestive tract after oral administration. It progresses through the digestive tract in an expected manner for mice: localized primarily in the stomach and intestine within the first two hours and within the colon between the second and fourth hour after administration. After 24 h, the polymer has been successfully eliminated from the digestive system. Merged images are represented (fluorescence in color and bright field in black and white).

1. **Biodistribution study with Cyanine 5.5 and gadolinium-labelled Chitosan@DOTAGA.** To study the elimination of Chitosan@DOTAGA, the polymer was labelled with Cyanine 5.5 and Gd^3+^ and was administered orally (one dose, 10 mg kg^−1^ of a 10 g L^−1^ of Chitosan@DOTAGA mixture solution). Mice were sacrificed at different time points (1, 2, 4 and 24 h) and organs collected for ex vivo analysis. Time points were chosen according to previous studies considering that mice have a total gastrointestinal transit time of about 6 h and that the majority of the intestinal content is located in small intestines and cecum after 3 h^36^. Ex vivo fluorescence imaging was first performed on the organs and semi-quantitative analysis performed as shown in Fig. 3 (as well as Fig. S8a–b). The stomach, intestine, and colon were the only organs where fluorescence was detected. There was no polymer found within the other organs: kidneys, liver, brain, spleen, heart, lungs, bone (skull), skin, and muscle. Chitosan@DOTAGA quickly passes through the stomach; before moving to the intestine (1–4 h) and then to the cecum and colon (2–4 h). The absence of alteration of the intestinal mucosa, which indicates the absence of toxicity and preservation of physiology and gastrointestinal integrity, was also verified by visual inspection. The polymer was completely eliminated in a time under 24-h. This is consistent with the gastrointestinal transit time of mice showing no undesirable adhesion of the compound to the GI tract.

To confirm these results, a quantitative analysis was performed on the organs by ICP-MS to determine the concentration of Gd^3+^ found within them, results are presented in Table 1. As seen with the fluorescence, the stomach, intestine, and colon were the only organs where gadolinium was identified and therefore where the polymer was found. Results showed a similar trend: Chitosan@DOTAGA quickly passes through the stomach; the intestine contains the majority of the polymer after 1 h; the colon contains the majority after 2–4 h. The polymer was completely eliminated in a time under 24-hours. Therefore, the Chitosan@DOTAGA compound has the potential to chelate lead or cadmium metal in the GI tract before being quickly eliminated, therefore preventing the absorption of these toxic metal.

2. **Effectiveness of Chitosan@DOTAGA.** To study the effectiveness of the polymer Chitosan@DOTAGA in scavenging lead and cadmium originating within foodstuff, the polymer was orally administered in mice daily for two weeks (daily dose, 10 mL kg^−1^ (~ 0.2 mL) at 5 g L^−1^ of polymer, 3.5 g L^−1^ NaCl) and studied against a control and a saline-treated (daily dose, 3.5 g L^−1^ NaCl) group. The experimental groups were exposed to rodent food contaminated with 7 mg kg^−1^ of cadmium and 50 mg kg^−1^ of lead while the control group was exposed to a non-contaminated food.

**Table 1:** Trend of gadolinium-marked Chitosan@DOTAGA over 24 h after oral administration in mice.

Organ	Sacrifice time (h)	Range of detected gadolinium (in Percentage of Administered Gadolinium)
Stomach	1	8.11–11.98
	2	1.09–5.83
	4	5.38–7.45
	24	< 1
Intestine	1	74.42–99.28
	2	< 1–3.56
	4	4.81–6.66
	24	< 1
Colon	1	2.08–14.12
	2	74.48–75.00
	4	46.53–67.53
	24	< 1

The administered amount of gadolinium was 8.75 µg by the oral administration of Chitosan@DOTAGA marked with gadolinium. 2 mice per time point. All other organs assess (liver, brain, kidneys, heart, lungs, spleen, muscle, bone (skull), and skin) reported < 1% of the administered dose at all of the time points, except for one liver which reported 6% after 1 h.

**Figure 4:**
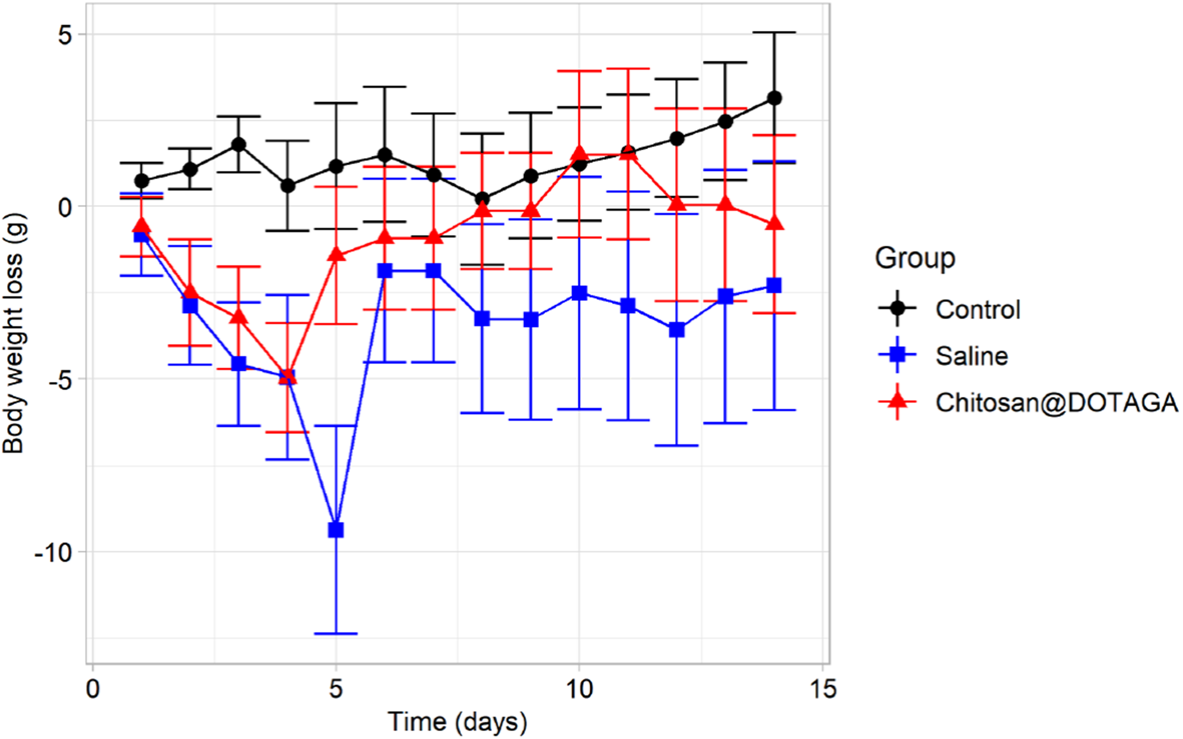
Relative change in body mass over 14 day experimental period. Over the course of 15 days, the trend in relative body weight compared to body weight at day 0 can be observed in the three population of mice. The saline-treated mice and Chitosan@DOTAGA-treated mice underwent an overall decrease in body weight in the first few days, but was quickly normalized.

All mice survived up to the final day of the study with no signs of toxicity and normal feeding, drinking, and overall behavior. Throughout the study, the weight of the mice was recorded. Both experimental groups (saline- and Chitosan@DOTAGA-treated) underwent a slight weight loss in the first few days, which was then normalized over the 14-day period. The relative change in body weight is presented in Fig. 4.

The gross pathology was assessed for all mice at the time of sacrifice. All of the saline-treated mice presented an increased gallbladder. Additionally, the bile was observed to be bright orange within the saline-treated mice, and sand in the bladder was detected in one mouse within this group. Instead, both control and Chitosan@DOTAGA-treated mice had the gallbladder of normal size and normally coloured (yellowish) bile. The color, shape, and structural features of all other internal organs were normal for the saline-treated mice. The Chitosan@DOTAGA-treated mice did not present any gross pathology abnormalities.

The blood metal concentration of the mice was assessed upon sacrifice to ensure a sufficient sampling volume for the analysis. Significantly higher blood lead and cadmium concentrations were seen among mice exposed to the contaminated rodent food compared to the mice exposed to uncontaminated rodent food, which is coherent with increased exposure, as presented in Table 2. On average, the Chitosan@DOTAGA-treated group presents a lower concentration of lead within their blood, although insignificant. This concentration compared to that of the saline-treated mice has a p-value of 0.093. Interestingly, two mice within the Chitosan@DOTAGA-treated group presented larger cadmium values that were not statistical outliers and therefore the reason in the average increase for this group. This may present evidence of individual differences in heavy metal exposure and elimination.

**Table 2:** The comparison of toxicity effects after lead and cadmium exposure (Saline; Chitosan@DOTAGA) evidencing the interest of an oral administration of Chitosan@DOTAGA.

Groups	Control	Saline	Chitosan@DOTAGA
Blood Lead (µg L^−1^)	68.2 ± 4.83	127.77 ± 18.32^c^	108.23 ± 18.13^b^
Blood Cadmium (µg L^−1^)	4.37 ± 1.24	7.75 ± 1.96^b^	16.67 ± 13.89^b^
ALP (U L^−1^)	259 ± 83	345 ± 183	201 ± 35
ALT (U L^−1^)	79 ± 55	65 ± 6	139 ± 136
AST (U L^−1^)	200 ± 88	201 ± 47	225 ± 78
RBC (10^12^ L^−1^)	8.39 ± 0.34	8.41 ± 1.05	8.82 ± 0.68
WBC (10^9^ L^−1^)	7.61 ± 1.98	4.42 ± 2.55^a^	6.43 ± 2.32
HGB (g L^−1^)	120.29 ± 5.68	116.33 ± 9.61	123.50 ± 2.43
HCT (%)	35.53 ± 1.36	35.68 ± 3.14	36.32 ± 1.13
PLT (10^9^ L^−1^)	1031.71 ± 274.34	846.67 ± 155.22	1254.83 ± 297
MCV (fL)	42.14 ± 2.19	42.67 ± 2.34	41.17 ± 2.48
MCH (pg)	14.34 ± 0.63	13.90 ± 0.82	14.10 ± 1.18
MCHC (g L^−1^)	336.50 ± 2.81	326.50 ± 2.59^d^	339.93 ± 10.83

*ALP* Alkaline phosphatase, *ALT* Alanine aminotransferase, *AST* Aspartate aminotransferase, *RBC* Red blood cell count, *WBC* White blood cell count, *HGB* Hemoglobin concentration, *HCT* Hematocrit, *MCV* Mean corpuscular volume, *MCH* Mean corpuscular hemoglobin, *MCHC* Mean corpuscular hemoglobin concentration.

All relations are compared to the control and are non-significant, except those defined: ^a^*p* < 0.05, ^b^*p* < 0.01, ^c^*p* < 0.001, ^d^*p* < 0.0001.

The liver function was assessed by liver enzyme activity assays. No significant differences in liver enzyme activity were observed between the control group and the saline-treated mice, and the experimental group did not present significant differences in liver enzyme activity when compared with both the control group and the saline-treated group, presented in Table 2.

The impact of lead and cadmium on hematological parameters were assessed and evidenced differences between the control and the saline-treated mice, the latter exhibiting decreased white blood cell count (WBC) and the mean corpuscular hemoglobin concentration (MCHC). The platelet count (PLT) and mean corpuscular hemoglobin (MCH) showed a decreasing trend within the saline-treated mice compared to the controls, although statistically insignificant. The other hematological parameters remained relatively stable between the groups: red blood cell count (RBC), hematocrit (HCT), and the mean corpuscular volume (MCV). With the treatment of Chitosan@DOTAGA, the WBC and MCHC were found to have similar values as the control group, where the polymer provides a “normalizing” effect, and a similar trend can be seen in the MCV concentration.

The histopathology of the organs of both experimental groups, saline- and Chitosan@DOTAGA-treated, exhibited injury/alteration patterns. The total scores for each organ are presented in Table 3. There was an observed increase in Kupffer cell accumulation, loci of lympho-histiocyte accumulation, substantial vessel congestion, and blood sinusoids dilation within the liver of the saline-treated mice which may evidence an inflammatory response and an altered blood supply. These were reduced with the treatment of Chitosan@DOTAGA, but still present. The saline-treated mice presented various alterations within the histology of the kidneys including signs of interstitial nephritis and glomerulo-nephritis and an altered blood supply, manifested by vessel congestion and capillary dilation. A loss of brush border was also observed in the tubular epithelium evidencing tubular injury within the kidneys. Of these alterations, the only one that persisted within the Chitosan@DOTAGA-treated mice was the dilation of the blood vessel with no signs of inflammation or tubular injury.

**Table 3:** Total histopathology scoring of mice organs after sacrifice.

Organ	Control	Saline	Chitosan@DOTAGA
Kidney	2	27	7
Liver	7	19	12
Spleen	1	6	8
Heart	1	8	5

Total score was calculated as a sum of individual scores. At least 5 random fields of view were observed for each magnification (× 100, × 400) from which at least 3 were quantified. N = 7 control, N = 6 Saline & Chitosan@DOTAGA.

Scoring and the assessed parameters for each organ detailed in the Supplementary Information, Table S6.

**Figure 5:**
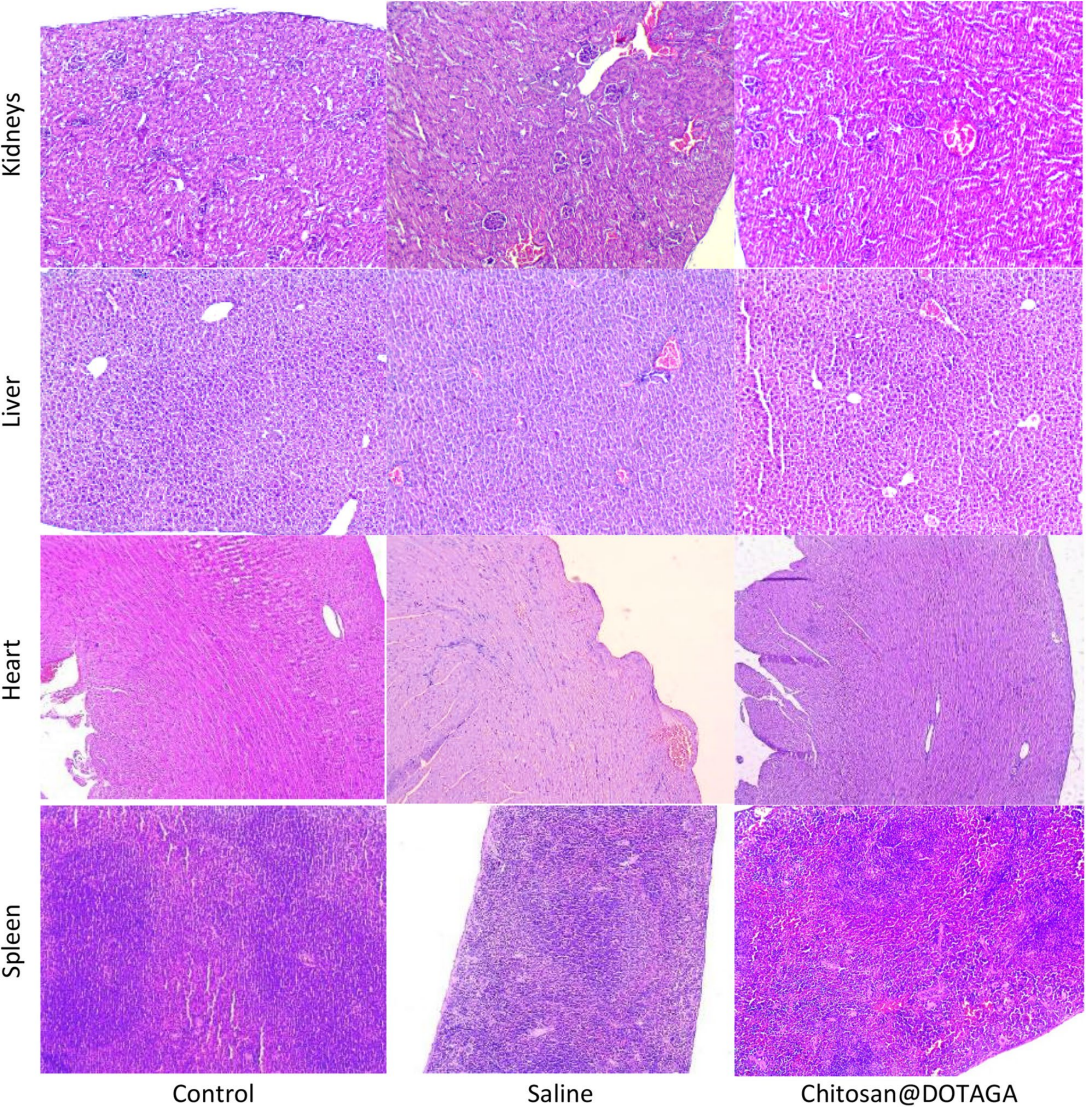
Histopathology of mice organs after sacrifice. Microphotographs of the kidneys, liver, heart, and spleen of the three experimental groups: control, saline-treated, and Chitosan@DOTAGA-treated. Magnification × 100, H&E. Kidney: interstitial nephritis, capillary dilation, loss of brush border (Saline-treated group), blood vessel dilation (Saline- and Chitosan@DOTAGA-treated groups); liver: Kupffer cell accumulation, lympho-histiocyte accumulation, vessel congestion, blood sinusoids dilation (Saline- and Chitosan@DOTAGA-treated groups); heart: blood vessel and capillary dilation (Saline- and Chitosan@DOTAGA-treated groups); spleen: marginal zone hyperplasia (Saline- and Chitosan@DOTAGA-treated groups), increased red pulp cellularity (Chitosan@DOTAGA-treated group).

In both saline- and Chitosan@DOTAGA-treated mice, minor inflammation within the heart can be observed with the occasional blood vessel and capillary dilation. The spleen of both treated groups demonstrated marginal zone hyperplasia, however, those treated with Chitosan@DOTAGA exhibited an increased red pulp cellularity, seen in Fig. 5.

## Discussion

Chitosan@DOTAGA can provide a new preventative and therapeutic strategy to combat heavy metal exposure from foodstuff and water. Chitosan is naturally occurring and is extracted from crustacean shells and fungi chitins with high purity, providing an innate biocompatibility. The re-acetylation and functionalization of chitosan with DOTAGA provides polyelectrolyte characteristics allowing this polymer’s solubility in water at a wider pH range than traditional chitosan polymers.

This study expands on the proposed probiotic by Zhai et al.^37^ which is used to prevent the absorption of cadmium within the digestive tract and combat its toxicity, with an easier to manufacture polymer while possessing a covalently bound chelating agent specific for heavy metal chelation. Housing the chelating agent on the polymer can eliminate the adverse effects that are reported with traditional chelation therapies, especially when orally administered like DMSA, by retaining the chelating agent in the target zone and reducing the required dose^27^.

Chitosan@DOTAGA was fully eliminated from mice (< 1% of administered dose) in less than 24 h after one oral administration as demonstrated by optical imaging and ICP-MS measurements of labelled polymer. Additionally, the polymer was fully retained within the digestive tract (stomach, intestines, and colon) and was not found in the other organs assessed. A similar pattern is therefore expected within a human model. This indicates that the Chitosan@DOTAGA compound would have the potential to chelate lead or cadmium metal in the GI tract before being quickly eliminated, therefore forcing the elimination of these toxic metals without entering the circulating blood.

The study of the effectiveness of Chitosan@DOTAGA mice, which was performed over a longer time frame, provided more results about its impact on body weight, blood concentration of lead and cadmium, liver enzyme activity, hematology, and histopathology of the organs. Additionally, it provides insights on the effectiveness and benefit that a daily, 1 mg treatment of Chitosan@DOTAGA in mice (i.e. 40 mg kg^−1^) can provide against the toxic effects of 7 mg kg^−1^ cadmium and 50 mg kg^−1^ lead within foodstuff.

The fluctuation of the mice body weights among the two experimental groups who received the contaminated rodent food compared to the control group were similar and provides evidence that these fluctuations are not due to the administered treatments, rather due to the exposure to lead and cadmium. This provides some general insight on the impact of contamination of lead and cadmium within foodstuff on body weight and is in agreement with previous animal studies^38–40^.

With no observed wounds or lesions, normal intestinal conditions, and normal fecal masses, it can be concluded that the oral administration of Chitosan@DOTAGA does not pose problems for the digestive tract and function.

The control mice, who were never exposed to lead and cadmium, presented pre-existing blood concentrations of lead and cadmium highlighting the importance of this study. Various studies show that the general population exhibits lead and cadmium within their blood even without major sources of exposure in their daily life^41,42^. Even mice are susceptible to this environmental exposure and present a significant blood lead concentrations of lead, which can lead to absorption and organ accumulation. There is no safe exposure and these metals can be observed even in laboratory animals^5,43^.

Understandably, the blood concentration of lead and cadmium within the saline-treated group were significantly higher than the control mice. Although not statistically significant, the Chitosan@DOTAGA-treated mice presented a decreased blood concentration of lead when compared to the saline-treated mice (*p* = 0.09309), which is approaching statistical significance, however this decreasing trend was not observed for the blood concentration of cadmium which could be attributed to its lower concentration when compared to lead. These results provide some preliminary evidence of improved heavy metal elimination within the Chitosan@DOTAGA-treated mice.

The average enzyme activity of ALT and AST within the control mice of this study were similar to previously reported levels within healthy mice^44,45^. The ALP activity within the control mice was higher than previously reported levels within healthy mice^46^. Previous studies employing chronic lead and cadmium exposure in rats or mice show significant changes in liver enzyme activity after longer timeframes^38,39^. Within this study detailed in this paper, two weeks of exposure does not present significant differences in ALT, ALP, and AST activity. Further, the activity of these liver enzymes remained consistent in the Chitosan@DOTAGA-treated group, evidencing the safety of this polymer with no negative impact on the liver function. Histopathological exploration provided evidence of liver inflammation within the saline-treated mice due to their exposure to lead and cadmium, which is in agreement with the findings of Andjelkovic et al.^47^. The treatment of Chitosan@DOTAGA minimized the inflammation within the liver to a lower prevalence. The constant liver enzyme activities could be explained by the high capacity of liver function, but the minimized inflammation can be attributed to the protective effect of the Chitosan@DOTAGA.

The kidneys were the organ which suffered the most within this study, evidencing signs of inflammation, altered blood supply, and direct tubular injury, which is all in agreement with previous findings and nephrotoxic effects of lead and cadmium^48,49^. Within the mice treated with Chitosan@DOTAGA, the kidneys exhibited no signs of inflammation or tubular injury. This provides strong evidence of the protective effect of Chitosan@DOTAGA on the kidneys within populations exposed to heavy metals.

The heart showed minor pathological changes that were similar to those seen within the other organs: inflammatory and signs of altered blood supply. These changes persisted even within the Chitosan@DOTAGA-treated mice. This may be explained by the strong epidemiological consequences of lead and cadmium on the cardiovascular system, which have been studied extensively on a long-term scale^50–52^. This dose of Chitosan@DOTAGA, although efficient in protecting other organs, may not be enough to prevent these cardiovascular effects.

The marginal zone hyperplasia observed within the spleen could serve as an indicator of the global inflammatory response directly related to the heavy metal exposure, which is in agreement with histopathological findings in the other organs. The increased red pulp cell count within Chitosan@DOTAGA-treated mice may be evidence of either an altered blood supply, which is in agreement with the other histopathological observations of mice organs made within this study, or an intensification of the hemopoiesis suffering due to heavy metal exposure^53^. Indeed, HGB and MCHC were decreased in saline-treated mice (significantly or as a trend) in this study, and Chitosan@DOTAGA application caused their reverse to control values and even exceeding those. Thus, such changes in spleen could be considered as a compensatory reaction. Nevertheless, both explanations can be directly associated with heavy metal exposure.

The hematological impact of lead and cadmium has been shown to vary based on the exposure level and whether these metals are combined or alone, but literature agrees that a chronic exposure to these metals has strong implications on the hemoglobin (HGB), red blood cell count (RBC), white blood cell count (WBC), hematocrit (HCT), mean corpuscular volume (MCV), mean corpuscular hemoglobin (MCH), mean corpuscular hemoglobin concentration (MCHC), and platelet count (PLT). Although lead and cadmium have been shown to affect the MCHC in an opposite trend, it is generally understood that exposure to these metals are associated to decreased levels/concentrations of these blood parameters^38–40^.

These observed trends can be attributed to the toxic effect of lead and cadmium on cell metabolism, displacement and disruptions of oligo-metals like calcium, inhibition of certain metabolic enzymes, and red blood cell affinity and destruction^6,11,13,54^. Lead and cadmium invoke anemia symptoms which can be evidenced by decreasing RBC, HGB, and HCT in long-term exposure situations^40^.

This short-term study shows a significant decrease in WBC and MCHC and decreasing trends in HGB and PLT within the mice that were exposed to contaminated food compared to those who were not exposed to contaminated food. These are preliminary signs of heavy metal poisoning, and over time, a continual decline of all other assessed blood parameters is expected if heavy metal poisoning continues.

Interestingly, the oral administration of Chitosan@DOTAGA maintains normal WBC, MCHC, PLT, and HGB levels which present no statistical differences from the control mice evidencing a remarkable preventative effect of this treatment against the heavy metals. This is also in agreement with the histopathology results showing an intensification of hemopoiesis due to the simple exposure to heavy metals.

This study provides evidence of the safety and facility of an oral administration of the polymer Chitosan@DOTAGA in mice while also displaying a protective effect from adverse effects caused by lead and cadmium within foodstuff. From these results, a human application seems feasible to protect against the toxic effects of lead and cadmium. Based on published heavy metal exposure within humans, it is anticipated that a dose around 100 mg of Chitosan@DOTAGA will provide real-time protective effects. Further regulatory studies will be planned to assess if this value can be used in human without chronic toxicity and impact on oligo-metals.

## Methods

### Polymer synthesis and characterization

*General synthesis & characterization* The Chitosan@DOTAGA polymer presented in this paper for the orally administered supplement was developed by MexBrain (patent “Polysaccharide Comportant un Groupement Chelatant Soluble…” Submission Number 1000503304, 29/07/2021)^55^. The synthesis of Chitosan@DOTAGA is performed using chitosan extracted from Alaska snow crabs with a weight-average molar mass (M_w_) of 2.583 × 10^5^ g mol^−1^ and a number-average molar mass (M_n_) of 1.323 × 10^5^ g mol^−1^. The characterization of the polymer is performed with techniques including high performance liquid chromatography (HPLC) and Nuclear Magnetic Resonance (^1^H NMR).

Detailed by Natuzzi et al.^35^, the synthesis is performed in a water and 1,2-propanediol environment with a mildly acidity in which acetic anhydride is added in molar ratios for specific acetylation of the chitosan polymer and DOTAGA anhydride for the grafting of DOTAGA. The chitosan was purchased from Matexcel (Bohemia, NY, USA, reference number NAT-0030), the DOTAGA anhydride was purchased from CheMatech (Dijon, France), the 1,2-propanediol and acetic anhydride were purchased from Sigma Aldrich (Saint-Quentin-Fallavier, France), and the acetic acid was purchased from VWR (France).

The NMR characterization evidences that 28% of the chitosan monomers are acetylated, and in conjunction with the HPLC–UV data, 7% of the monomers house a molecule of grafted DOTAGA while 65% of monomers remain de-acetylated in the form of Glucosamine (GlcN). This formulation is soluble in water ranging from pH 2–9 with and without salts and provides a stability up to 50 gL^−1^^35^.

The complexation coefficients for DOTA and some metals are listed in the Supplementary Information^31,32^. Due to DOTAGA’s similar structure, the chelation is expected to follow a similar pattern.

### Characterization of lead and cadmium chelation by Chitosan@DOTAGA

*Materials and chemicals* The acetate buffer used as the eluent for the HPLC is composed of 0.1 M acetic acid and 0.1 M ammonium acetate in water. The acetic acid (CAS 64-19-7) and MS-grade ammonium acetate (CAS 631-61-8) were purchased from Sigma Aldrich (Saint-Quentin-Fallavier, France). The standard ICP-MS metal solution was provided by SCP Science (Quebec, Canada) as a custom formulation with 10 µg mL^−1^ of Pb, Cd, Al, Mn, Zn, Cu in 4% HNO_3_. The standard Pb solution was provided by SCP-Science: ICP Standard Pb 1000 µg mL^−1^ 140-051-821 SCP Science. The standard Cd solution was provided by SCP-Science: ICP Standard Pb 1000 µg mL^−1^ 140-051-481 SCP Science.

*Preparation of solutions* Chitosan@DOTAGA was dissolved in MilliQ water to attain a concentration of 10 g L^−1^. The samples were created using 0.1 M acetate buffer as the matrix with an addition of Chitosan@DOTAGA and/or the metals. The final concentration of polymer within the sample was 0.1 g L^−1^ and the final concentration of the metals ranging from 0.5 to 200 µg L^−1^.

*Experimental protocol* PerkinElmer Flexar High Performance Liquid Chromatography (HPLC) in series with PerkinElmer NexION 2000 ICPMS were used to analyze the chelation of lead and cadmium by Chitosan@DOTAGA. The samples were analyzed using a Polysep GFC-P 4000 series column with a 10 µL injection volume, a flow rate 0.4 mL min^−1^, 0.1 M MS grade acetate buffer, 270 PSI, 40-min acquisition, in standard ICP mode for the analysis of ^114^Cd and ^208^Pb.

### In vivo oral administration study of Chitosan@DOTAGA in mice

1. **Biodistribution Study of gadolinium-labelled and Cyanine5.5-labelled Chitosan@DOTAGA.** *Chemicals & Equipment.* Gadolinium (III) chloride hexahydrate (GdCl_3_ × 6 H_2_O, CAS 13450-84-5, 99.9%) was purchased from Sigma Aldrich (Saint-Quentin-Fallavier, France). Cyanine5.5-NHS ester (CAS 2375105-86-3) was purchased from Sigma Aldrich (Saint-Quentin-Fallavier, France).

Gadolinium-labelled Chitosan@DOTAGA was synthesized, purified, and characterized. The synthesis was performed using a previously synthesized and characterized Chitosan@DOTAGA (batch AERIS 01-12, 7.2 g L^−1^, 1.5 L). 302.8 mg of the gadolinium salt was added and stirred at room temperature for 1 h. The pH was then adjusted to 6.0 ± 0.1 and the solution was stirred at 60 °C for 48 h. The polymer was purified by tangential filtration to eliminate the non-chelated gadolinium. The polymer was then analyzed by inductively coupled plasma mass spectrometry (ICP-MS) to analyze the concentration of chelated gadolinium per gram of polymer, determined to be 8.75 mg Gd^3+^ per gram of Chitosan@DOTAGA.

Cyanine5.5-labelled Chitosan@DOTAGA was synthesized, purified, and characterized. The synthesis was performed using a previously synthesized and characterized Chitosan@DOTAGA. Cyanine5.5-NHS ester was dissolved in DMSO with a final concentration of 25 g L^−1^. 70 µL of this solution was added and stirred at room temperature for 4 h with ample protection from the light. The polymer was purified using a Vivacell 100 Centrifuge tube with an internal membrane of 100 kDa. The solution was centrifuged at 2000 rpm for 15 minute cycles until reconcentrated to a final concentration of 10 g L^−1^. The polymer was then analyzed by HPLC-UV at 640 nm to visualize the Cyanine 5.5. The amount of grafted Cyanine5.5 was determined to be 7.5 mg Cyanine 5.5 per gram of Chitosan@DOTAGA.

A mixture solution of the gadolinium-labelled Chitosan@DOTAGA and Cyanine 5.5-labelled Chitosan@DOTAGA (total concentration 10 g L^−1^ (5 g L^−1^ gadolinium-labelled and 5 g L^−1^ Cyanine5.5-labelled), no salt, sterilized) were then used for the biodistribution study.

*Animals & dosing* All animal experiments were approved by the local animal ethics of University Claude Bernard Lyon 1 and carried out in accordance with current French and ARRIVE guidelines (APAFIS#30771-2020102608418246 v4). Healthy female C57Bl/6 mice (~ 10 weeks) were used for the experiment. At the beginning of the experiment, one dose (10 mL kg^−1^, 10 gL^−1^ mixture) of labelled mixture of Chitosan@DOTAGA was orally administered to the mice with a final administration of 8.75 µg Gd^3+^ and 7.5 µg Cyanine5.5.

At scheduled time points (1, 2, 3, 4, and 24 h), 2 mice were anesthetized with isoflurane and sacrificed through cervical dislocation. Organs (stomach, intestines, colon, bone from the leg, skin, muscle, blood, urine, kidneys, liver, brain, spleen, heart, lungs) were harvested.

Ex vivo *optical imaging* Ex vivo fluorescent measurements were performed immediately after organ collection to determine the accumulation of the dye and organs were then stored at − 80 °C until further analysis. Fluorescence images as well as bright-field images were acquired via a back-thinned CCD-cooled camera ORCAIIBT-512G (Hamamatsu Photonics Deutschland GmbH, Herrsching am Ammersee, Germany) using a coloured glass long-pass RG 665 filter (Melles Griot, Voisins les Bretonneaux, France). Optical excitation was carried out at 633 nm, and the emission wavelength was detected at 680 nm. Exposure time was set at 30s for optical imaging and 0.05 s for bright field imaging.

Images were analyzed using FIJI ImageJ software with Java version 1.8.0_172. The same brightness and contrast parameters were applied to all images to obtain comparable results. The bright field and fluorescence images were then merged to obtain the results seen in Fig. 3. The quantification of fluorescence signal in each organ was performed following the protocol described previously by Labno and presented in S8^56^.

Ex vivo *Gd*^*3*+^ *quantification analytical procedures* A Multiwave 5000 Anton Paar microwave and associated equipment was used for organ digestion with nitric acid (HNO_3_, CAS 7697-37-2, 69%) purchased from Carl Roth (France). The samples were analyzed using a Perkin Elmer NexION 2000 ICP-MS. A standard calibration was created using gadolinium standard purchased from SCP Science (Article number 140-061-641).

2. **Effectiveness study of Chitosan@DOTAGA.** *Animals.* This study design, animal selection, handling, and treatment were all in accordance with ARRIVE and Animal Care guidelines (Palladin Institute of Biochemistry of the NASU Animal Care and Use Committee protocol #1 dated Jan 10 2022). All mice are drug and test naïve. The mouse strain C57Bl6 was used for this study. This strain of mice is commonly used for toxicity and efficiency studies, and therefore is an appropriate choice for this study. Animals had free access to standard or enriched rodent chaw and free access to boiled tap water. A seven-day acclimation period was respected before the study began. Group randomization was based on body weight.

All in vivo protocols are in adherence with the European Convention for the Protection of Vertebrate Animals used for Experimental and other Scientific Purposes.

*Chemicals & equipment* Cadmium chloride (CdCl_2_ × H_2_O, CAS 654054-66-7, 99.995%) was purchased from Sigma Aldrich (Schnelldorf, Germany) and lead acetate (Pb(CH_3_COO)_2_ × 3H_2_O, CAS 6080-56-4, 99.995%) was purchased from ALFA AESAR/Thermo Fischer Scientific (Karlsruhe, Germany). These salts were used in creation of the experimental feed. Micros ABX VET (Horiba Medical, Grabels, France), Selectra ProM (ELITech Group, Puteaux, France), and a Shimadzu ICPE 9820 (Shimadzu Corporation, Tokyo, Japan) were employed as analytical tools within this study for hematology, biochemistry, and spectrometry, respectively.

*Analytical procedures* The activity of AST, ALT and ALP enzymes (liver functional activity markers) were assessed in blood serum, using commercial kits according to the manufacturer’s instructions (Cormay). Cadmium and Lead were detected in whole blood by inductively-coupled plasma optical atomic emission spectrometry method after samples microwave mineralization in concentrated nitric acid solution with added H_2_O_2_. Hematology analysis was performed by Hematology Analyzer Micros ABX VET (Horiba Medical, France). Histopathology analysis was performed on hematoxylin–eosin-stained slides obtained from formalin-fixed paraffin embedded kidney, liver, spleen and heart samples in a blinded manner. The analyzed parameters for each organ and scoring are detailed in the Supplementary Information and Table S6. The scoring was based on the percent of the visual field presenting the parameter in question, ranging from 0 to 6 for necrosis and hemorrhage and 0 to 3 for all others. The final score for each parameter was calculated as a sum of three analyzed sections on the organ. The scores presented in Table 3 reflect the sum of all parameters for the given organ.

*Dose levels, group division, and sampling* Three experimental groups were defined for this study: control group (Group 1, 14 mice), saline-treated group (Group 2, 12 mice), Chitosan@DOTAGA-treated group (Group 3, 12 mice). Groups 2–3 were fed rodent chaw enriched with cadmium and lead salts: 7 mg kg^−1^ cadmium and 50 mg kg^−1^ lead, while Group 1 was fed standard rodent chaw. Groups 2–3 were treated by oral administration of 10 mL kg^−1^ of the corresponding test compound: Group 2 received 3.5 g L^−1^ NaCl in water, Group 3 received 5 g L^−1^ Chitosan@DOTAGA in solution with 3.5 g L^−1^ NaCl.

Necropsy with blood and tissue samples collection and fixation (liver, kidney, spleen, heart) were performed at the terminal sacrifice, on the 15th day. Hematology and histopathology analyses, biochemical (ALT, AST, ALP) analyses, and blood analyses for cadmium and lead were performed on the 15th day of following the sacrifice.

*Group characteristics* Mice were 11–12 weeks old, the initial body weight ranged from 17 to 29 g. Average body weight across all experimental groups was 23.1 g (SD = 2.7 g, CV = 11.6%). Body weight was measured every day between 8–9 AM.

*Frequency & duration of treatment* Animals were treated with Chitosan@DOTAGA or a saline solution for 14 days beginning 24 h after the first exposure to the diet. The treatments were orally administered once per day between 8–9 AM (10 mL kg^−1^).

*Statistical analysis* Outliers were removed by respecting the 1.5xIQR statistical rule for each data set.

Before performing statistical analysis, the normality and homoscedasticity of the samples were assessed using the Shapiro test and the Bartlett test. According to these tests, either a t-test or a Kruskal–Wallis test was applied to assess the statistical significance between groups. All analyses were performed on RStudio v4.0.3 using the stats package V3.6.2.

## Supplementary Information

Supplementary Information 1.

Supplementary Information 2.

## Data Availability

All data generated and analyzed during the in vivo studies are included in this paper and its supplementary information. The datasets corresponding to the characterization of the polymer are available from the corresponding author on reasonable request.
