# Supplementary material for: Combating lead and cadmium exposure with an orally administered chitosan-based chelating polymer
Source: Sci Rep. 2023 Feb 7;13:2215. doi: 10.1038/s41598-023-28968-4 (PMC9905611; doi:10.1038/s41598-023-28968-4)
Supplement: Supplementary file 1 — Supplementary Information 1. [file 41598_2023_28968_MOESM1_ESM.docx]

**SUPPLEMENTARY MATERIALS**

Table S0. Complexation Coefficients for DOTA from Anderegg et al., 2005.^1^

| **Metal** | **Logβ  M+DOTA = [M(DOTA)]** |
| --- | --- |
| Pb^2+^ | 22.69 |
| Cd^2+^ | 21.3 |
| Cu^2+^ | 22.3 |
| Fe^3+^ | 29.4 |
| Ni^2+^ | 20.03 |
| Co^2+^ | 20.27 |
| Mn^2+^ | 20.0 |

**Efficiency Study of Chitosan@DOTAGA**

***Housing Conditions of Animals.***

| Hygienic level: | Good conventional |
| --- | --- |
| Type of animal cages: | Polypropylene bottoms with stainless steel wire mesh lids |
| Cage size: | H x W x D: 18.0 x 25.5 x 40.0 cm |
| Cleaning: | By changing the bedding twice a week |
| Number of animals per cage: | 6-7 |

*Environmental conditions*

| Air exchange: | 15-20 times/hour |
| --- | --- |
| Temperature: | 22 ± 3 ^0^C |
| Relative humidity: | 40 – 70 % |
| Lighting: | natural |

*Feed.* Mice were given free access to standardized or customized (enriched with Cd and Pb salts) rodent diet according to the study design.

*Drinking.* Mice had free access to boiled tap water.

***Acclimation Period of Animals.***

A required acclimation period of seven days was required upon arrival of mice. Cages housed 6-7 animals during this period. All animals were under observation and only the animals presenting no clinical signs of illness were chosen for this study.

***Randomization of Animals.***

Animals were randomized based on body weight and assigned to groups prior to the first day of the experiment. Animals were housed together based on their experimental group.

***Anesthesia, Histology Reagents, and Enzyme Analysis.***

2,2,2-tribromoethanol and 2-methyl-2-butanol from Sigma Aldrich were used as anaesthetic reagents. 37% formaldehyde (Ukrorgsynthesis Ltd, Ukraine), 95% Ethanol (Ukrzoovetprompostach, Ukraine), Paraffin Type 6 (Thermo Scientific), Chrolorform (Ukrorgsynthesis Ltd, Ukraine), Hematoxylin and Eosin Y (Sigma Aldrich) were used as histology reagents.

Aspartate aminotransferase (AST). The principle of the method is the ability of aspartate aminotransferase to catalyze the interconversion of aspartate and α-oxoglutarate to oxaloacetate and glutamate. The rate of absorbance change at λ=340 nm is directly proportional to ASAT activity.

Alanine aminotransferase (ALT). The principle of the method is the ability of alanine aminotransferase to catalyze the interconversion of L-alanine and α-oxoglutarate to pyruvate and glutamate. The rate of absorbance change at λ=340 nm is directly proportional to Alanine aminotransferase activity.

Alkaline phosphatase (ALP). Kinetic method with 2-amino-2-methyl-1-propanol. The method is based on the enzyme ability to catalyze the interconversion of 2-amino-2-methyl-1-propanol and p-nitrophenylophosphate to 4-nitrophenol and 2-amino-2-methyl-1-propanol phosphate. The rate of of 4-nitrophenol formation measured colorimetrically is directly proportional to the ALP activity.

***Observations & Examinations of Animals: Lethality.***

The animals were monitored for mortality immediately after the first compounds administration and daily during the observation period.

***Observations & Examinations of Animals: Clinical Signs.***

Toxic effects of compounds were evaluated during the study (14 days) by visual observations according to the standard procedures and FELASA recommendations.^2^ The external state of the skin and fur, eyes, and mucous membranes, as well as the respiratory system, posture, and changes in spontaneous activity were evaluated. The presence of tremor, convulsions, salivation, and diarrhea was considered as well. Clinical signs were tested during the study with scores: general appearance (normal – 0; unnatural posture/hunched pose – 2; emaciation– 3); hypo/hyperkinesia (absent – 0; decrease/increase activity – 1; drowsiness /aggression – 2; unresponsive to extraneous activity and provocation – 3); movement activity (normal – 0; dysbasia/circling – 1; tremor – 2; convulsions, limb paralysis – 3); respiration alterations (absent – 0; deep/heavy/rapid/shallow – 1; respiratory arrest – 5); skin/coat injuries (absent – 0; redness – 1; wounds – 2; abscess – 3; necrosis – 4); piloerection (absence – 0; presence – 1); eyes conditions alterations (absent – 0; pale/clouded eyes/tearing – 1; sunken/inflamed/half-closed eyes – 2; closed eyes, does not open on touch – 3); exudation (absent – 0; ptyalism/nasal exudation – 2); defecation changes (absent – 0; abdomen abnormally enlarged – 1; loose stool – 1; constipation – 2; diarrhea – 3; defecation with blood – 4); oedema/ alterations including the site of administration (absent – 0; oedema/other changes at the site of administration – 1; not at the site of administration – 2); body temperature (normal – 0; increased/decreased - 2); vocalization (absence – 0; occasional – 1; consistent - 2); death of the animal – 10. Observations were performed immediately after the first administration, and once a day during the observation period.

***Observations & Examinations of Animals: Blood Collection & Euthanasia.***

Terminal bleeding and euthanasia were performed on the 15th days of the study for all groups. Animals were injected IP with 2,2,2-tribromoethanol (150 mg/kg). The blood was collected from orbital sinuses into dry microtainers without anticoagulant. For hematological study, 50 µl of fresh blood was transferred in special vacutainers.

The tubes with collected blood were stored at room temperature for 15-60 min after blood drawing before centrifugation to form a fibrin clot, and then were centrifuged at 9000 rpm for 20 minutes at +4°C. The collected serum was subjected to biochemical analysis and Cd/Pb detection at the same day.

Euthanasia was performed after terminal bleeding using cervical dislocation. All procedures were in accordance with the OECD recommendations.^3^

***Observations & Examinations of Animals: Gross Pathology.***

Gross pathology was carried out to detect and record abnormal external findings and macroscopic alterations in internal and external organs. The following organs and parameters were investigated:

- Coat and general external condition of the animals according to the 3-level ranking: good, tolerable and poor.
- Blood color in the vessels. Since the evaluation was done immediately after euthanizing the mice, blood color did not differ between the animals and therefore was not mentioned in the report.
- Intestines. Presence and amount of gas and faeces in the intestines.
- Internal organs condition. Color, shape, consistency, structural peculiarities, and size of the organs (kidneys, spleen, liver, heart) were investigated

***Histopathology of Animals.***

Formalin-fixed paraffin-embedded hematoxylin-eosin (H&E)-stained films of kidney, liver, spleen and heart were analysed under the light microscope.

The following parameters were evaluated for **kidney** samples: 1) glomerulus shrinkage/capsule space dilation, 2) tubular epithelium flattening, 3) epithelial cell vacuolation, 4) epithelial cell desquamation/tubular epithelium loss, 5) loss of brush border, 6) tubular atrophy, 7) tubular dilation, 8) loss of tubules, 9) eosinophilic cast deposition, 10) necrosis loci, 11) interstitial nephritis as lympho-histiocyte infiltration of tubular epithelium and interstitium, 12) glomerulonephritis as lympho-histiocyte infiltration of renal capsule, 13) vessel dilation, 14) hemorrhage, 15) fibrosis as connective tissue accumulation, 16) tubular hyperplasia as the sign of tubular cells proliferation and tubule regeneration. Traits # 1-8 evidence the tubular state, traits # 9-12 - necrotic and inflammatory signs, traits #13-14 – blood vessels state, traits #15-16 – tissue regeneration (normal or abnormal).

The following parameters were evaluated for **spleen** samples: 1) lymphoid hypoplasia as reduced white pulp, 2) lymphoid atrophy, 3) lipid accumulation, 4) pigmentation, 5) fibrosis as connective tissue accumulation, 6) necrosis, 7) hemorrhages, 8) granulomas, 9) red pulp hyperplasia, 10) white pulp hyperplasia, 11) megakaryocytosis, 12) marginal zone hyperplasia.

The following parameters were evaluated for **liver** samples: 1) lipid dystrophy, 2) vessel congestion/dilation, 3) blood sinusoids dilation, 4) lympho-histiocytes accumulation loci, 5) Kupffer cell diffuse accumulation, 6) ground-glass hepatocytes, 7) eosinophilic alteration, 8) basophilic alteration, 9) necrotic hepatocytes, 10) apoptotic hepatocytes, 11) fibrosis as connective tissue accumulation, 12) necrosis, 13) polyploid cells, 14) glycogen overload.

The following parameters were evaluated for **heart** samples: 1) vessel dilation/thrombosis, 2) blood capillary dilation, 3) inflammatory infiltrate, 4) cardiomyocyte disorganization as eosinophilia and loss of striation, 5) necrosis, 6) fibrosis as connective tissue accumulation, 7) fatty changes, 8) lipofuscinosis as lipofuscin depositions, 9) hemorrhage.

At least 5 random fields of view on every magnification (×100, ×400) of each tissue were analysed.
